# Combination of gene expression patterns in whole blood discriminate between tuberculosis infection states

**DOI:** 10.1186/1471-2334-14-257

**Published:** 2014-05-13

**Authors:** Adane Mihret, Andre G Loxton, Yonas Bekele, Stefan HE Kaufmann, Martin Kidd, Mariëlle C Haks, Tom HM Ottenhoff, Abraham Aseffa, Rawleigh Howe, Gerhard Walzl

**Affiliations:** 1 Armauer Hansen Research Institute, Addis Ababa, Ethiopia; 2 Department of Microbiology, Immunology and Parasitology, School of Medicine, College of Health Sciences, Addis Ababa University, Addis Ababa, Ethiopia; 3 Division of Molecular Biology and Human Genetics, DST/NRF Centre of Excellence for Biomedical Tuberculosis Research, MRC Centre for Molecular and Cellular Biology, Faculty of Medicine and Health Sciences, Stellenbosch University, Francie van Zijl Drive, P.O. Box 19063, 7505 Tygerberg, South Africa; 4 Department of Immunology, Max Planck Institute for Infection Biology, Berlin, Germany; 5 Centre for Statistical Consultation, Department of Statistics and Actuarial Sciences, University of Stellenbosch, Stellenbosch, South Africa; 6 Department of Infectious Diseases, Leiden University Medical Centre, Leiden, The Netherlands

## Abstract

**Background:**

Genetic factors are involved in susceptibility or protection to tuberculosis (TB). Apart from gene polymorphisms and mutations, changes in levels of gene expression, induced by non-genetic factors, may also determine whether individuals progress to active TB.

**Methods:**

We analysed the expression level of 45 genes in a total of 47 individuals (23 healthy household contacts and 24 new smear-positive pulmonary TB patients) in Addis Ababa using a dual colour multiplex ligation-dependent probe amplification (dcRT-MLPA) technique to assess gene expression profiles that may be used to distinguish TB cases and their contacts and also latently infected (LTBI) and uninfected household contacts.

**Results:**

The gene expression level of *BLR1, Bcl2, IL4d2, IL7R, FCGR1A, MARCO, MMP9, CCL19*, and *LTF* had significant discriminatory power between sputum smear-positive TB cases and household contacts, with AUCs of 0.84, 0.81, 0.79, 0.79, 0.78, 0.76, 0.75, 0.75 and 0.68 respectively. The combination of *Bcl2, BLR1, FCGR1A, IL4d2* and *MARCO* identified 91.66% of active TB cases and 95.65% of household contacts without active TB. The expression of *CCL19, TGFB1,* and *Foxp3* showed significant difference between LTBI and uninfected contacts, with AUCs of 0.85, 0.82, and 0.75, respectively, whereas the combination of *BPI, CCL19, FoxP3, FPR1* and *TGFB1* identified 90.9% of QFT^-^ and 91.6% of QFT^+^ household contacts.

**Conclusions:**

Expression of single and especially combinations of host genes can accurately differentiate between active TB cases and healthy individuals as well as between LTBI and uninfected contacts.

## Background

An effective immune response controls *Mycobacterium tuberculosis* (MTB) in the majority of infected individuals, and only 3-10% of those infected persons develop clinical disease and symptoms within the first two years after infection (primary tuberculosis, TB) while another 5% develop the disease later in life (reactivation TB) [1]. Defining the differences in the immune responses between those who control versus those who fail to control the infection is an important prerequisite for the development of interventions that will improve immune-mediated protection. Various studies have confirmed that genetic factors are involved in the disease and could be key for the different outcomes of MTB infection [2,3]. A recent study showed a significant difference in the type and magnitude of immune responses between UK and Malawi children against BCG. Th1 related cytokines were present at higher levels in the UK infants whereas abundances of innate proinflammatory cytokines, regulatory cytokines, interleukin 17, Th2 cytokines, chemokines and growth factors were elevated in the Malawi infants, possibly due to genetic but also environmental factors [4].

Apart from genetic factors lead to differences among individuals [3,5-10], environment-induced changes in gene expression occur during the dynamic interaction between the immune system and *M. tuberculosis *[11-14]. Therefore, assessing differential regulation of gene expression may help identifying biomarkers to distinguish the different MTB exposure outcomes. Recent studies have indicated that *Fc gamma receptor 1B (FCGR1B) *[14], combined with expression patterns of *FCGR1A (CD64), RAB33A* and *LTF* (lactoferrin) [12] and *CD3E, CD8A, IL7R, BLR1, CD19, FCGR1A, CXCL10, CD4, TNF, BCL2, MMP9, Foxp3, CASP8, CCL4, TNRFSF1A, CASP8, Bcl2 and TNF *[15] showed clear discriminatory power between TB and latent TB infection (LTBI). Expression of *RIN3, LY6G6D, TEX264*, and *C14orf2* genes identified active, cured, recurrent or LTBI [11]. Therefore, we analysed 45 genes targeting immune cell subset markers, T regulatory cell markers, effector T cell markers, apoptosis related genes and four housekeeping genes using a dual color multiplex ligation-dependent probe amplification technique (dcRT-MLPA) to assess gene expression profiles to distinguish between the different clinical groups. These markers were selected for gene expression profiling, as described in Joosten et al. [15].

## Methods

A total of 47 subjects (23 healthy household contacts and 24 microbiologically confirmed new smear-positive HIV negative pulmonary TB patients) attending Arada, T/Haimanot, Kirkos and W-23 health centres in Addis Ababa were recruited upon informed consent.

The diagnosis of active TB in the health centres was based on the national guidelines of at least two positive sputum smears for acid-fast bacilli (AFB) in three specimens collected from each patient as spot-morning-spot samples. All sputum samples from TB cases were cultured for mycobacteria and confirmed as MTB. We obtained ethical clearance from AHRI/ALERT Ethics Review Committee (P015/10) and National Research Ethics Review Committee (NRERC) (3.10/17/10).

QuantiFERON-TB Gold In Tube (QFT-GIT) test was used to detect LTBI as per the manufacturer’s instructions (Cellestis Limited, Carnegie, Victoria, Australia) [16]. Three ml venous blood was directly collected into three 1-ml QFT-GIT tubes (Cellestis, Australia); one negative control (Nil) tube containing only heparin, another tube containing phytohaemagglutinin (PHA) as positive control (Mitogen) and the third tube containing overlapping peptides representing the entire sequences of ESAT-6, CFP-10 and TB7.7 (TB Antigen). The tubes were shaken vigorously and then incubated at 37°C for about 20 hrs. They were then centrifuged and plasma was harvested and frozen at -20°C until ELISA was performed. The level of IFN-γ was measured using the QFT ELISA kit (Cellestis, Australia). The ELISA readout and data interpretation were carried out using the QFT software (Version 2.50, Cellestis, Australia). As recommended by the manufacturer, a positive test for MTB infection was considered if the IFN-γ difference was ≥0.35 IU/ml (TB antigens–Negative control). The result of the test was considered indeterminate when an antigen-stimulated sample was ≤ 0.35 IU/ml (TB antigens–Negative control) if the value of the positive control was less than 0.5 IU/ml (Positive control–Negative control).

### Blood collection and RNA extraction

Venous blood was collected into PAXgene Blood RNA tubes and RNA extraction performed following the manufacturer’s instructions (PAXgene Blood RNA Kit, PreAnalytiX, QIAGEN) [17]. Briefly, blood containing tubes were centrifuged at 3000 rpm for 10 min, supernatant discarded, pellet lysed and washed, followed by treatment with proteinase K and ethanol precipitation. To remove contaminating DNA, RNase-free DNase was added (QIAGEN, Germany), washed and finally the RNA was eluted with RNase-free water, concentration-quantified using a GeneQuant spectrophotometer (Amersham Biosciences, UK) and stored at -80°C until use.

### Dual colour multiplex ligation-dependent probe amplification (dcRT-MLPA)

dcRT-MLPA was done according to ref Joosten et al. [15]. First, cDNA was synthesised from RNA by using a RT primer mix and then denatured and incubated overnight with the mixture of customized probes to allow the probes to hybridize with the target genes. The two separate probes were then fused together using a ligase enzyme. The ligated probes hybridized with the target genes, were amplified. Finally the PCR product was separated by electrophoresis and the RNA expression levels were quantified by measuring the fluorescence intensity.

A set of probes was designed by Leiden University Medical Centre (LUMC), Leiden, The Netherlands, and comprised sequences for 45 genes targeting immune cell subset markers, T reg markers, effector T cell markers and apoptosis related genes and four housekeeping genes. Genes associated with active TB disease or protection against disease, as described in the literature, were included in the study. The list of genes for which a set of probes was designed is shown in Table 1.

**Table 1:** List of target genes for dual colour multiplex ligation-dependent probe amplification (dcRT-MLPA)

Bcl2	CD8α	IL4	RAB33
BLR1	CD14	IL4d2	SEC14L1
BPI	CD19	IL7R	SPP1
CASP8	CD163	IL10	TGFB1
CCL4	CTLA4	IL22RA1	TGFBR2
CCL13	CXCL10	LAG3	TNF
CCL19	FASLG	LTF	TNFRSF1A
CCL22	FCGR1A	MARCO	TNFRSF1B
CCR7	FOXP3	MMP9	TIMP2
CD3ϵ	FPR1	NCAM1	TNFRSF18
CD4	IFN γ	RAB13	Reference genes
	IL2Rα	RAB24	ABR, β2M, GAPDH, GUSB

After completion of the dcRT-MLPA reaction, amplified products were analysed with an ABI-310 capillary sequencer in GeneScan mode (Applied Biosystems). The data from the sequencer were analysed using the GeneMapper software. Further analysis was done using Microsoft Excel spread sheet software. Finally data were normalised by selecting one of the housekeeping genes, which was most stably expressed across the evaluated samples (ABR, GUSB, GAPDH or B2M). The coefficient of variation was calculated to determine which reference gene was most stably expressed across the evaluated samples. GAPDH was selected and all samples were normalized over GAPDH. A peak area of 200 for signals was assigned as threshold value for noise cut off in GeneMapper. The relative peak size of the product from the probe recognition sequence was compared with the relative peak size of the product from a control.

### Statistical analysis

The data were analyzed using Graph Pad Prism software, version 4.0 (La Jolla, CA 92037 USA) and STATISTICA software, version 10, Statsoft (Ohio, USA). Nonparametric Mann–Whitney U tests were performed to find the significance of the observed differences. Best subsets discriminant analysis (GDA) and receiver operator characteristic (ROC) curve analysis were used to evaluate the predictive abilities of combinations of biomarkers and to generate cut off values for differentiating between MTB infection states (described in [18]). A p value less than 0.05 was considered as statistically significant.

## Results

We enrolled 24 subjects with culture confirmed HIV^-^ active tuberculosis TB and 23 HIV^-^ household contacts comprising 12 QFT-GIT^+^ and 11 QFT-GIT^-^ household contacts. The mean age of TB patients was 31.6 ± 1.4 and 46.5% of the participants were females. The mean age for household contacts was 28.3 ± 2.3 and 47.6% of household contacts were females.

### Gene expression of TB patients and household contacts

**Figure 1:**
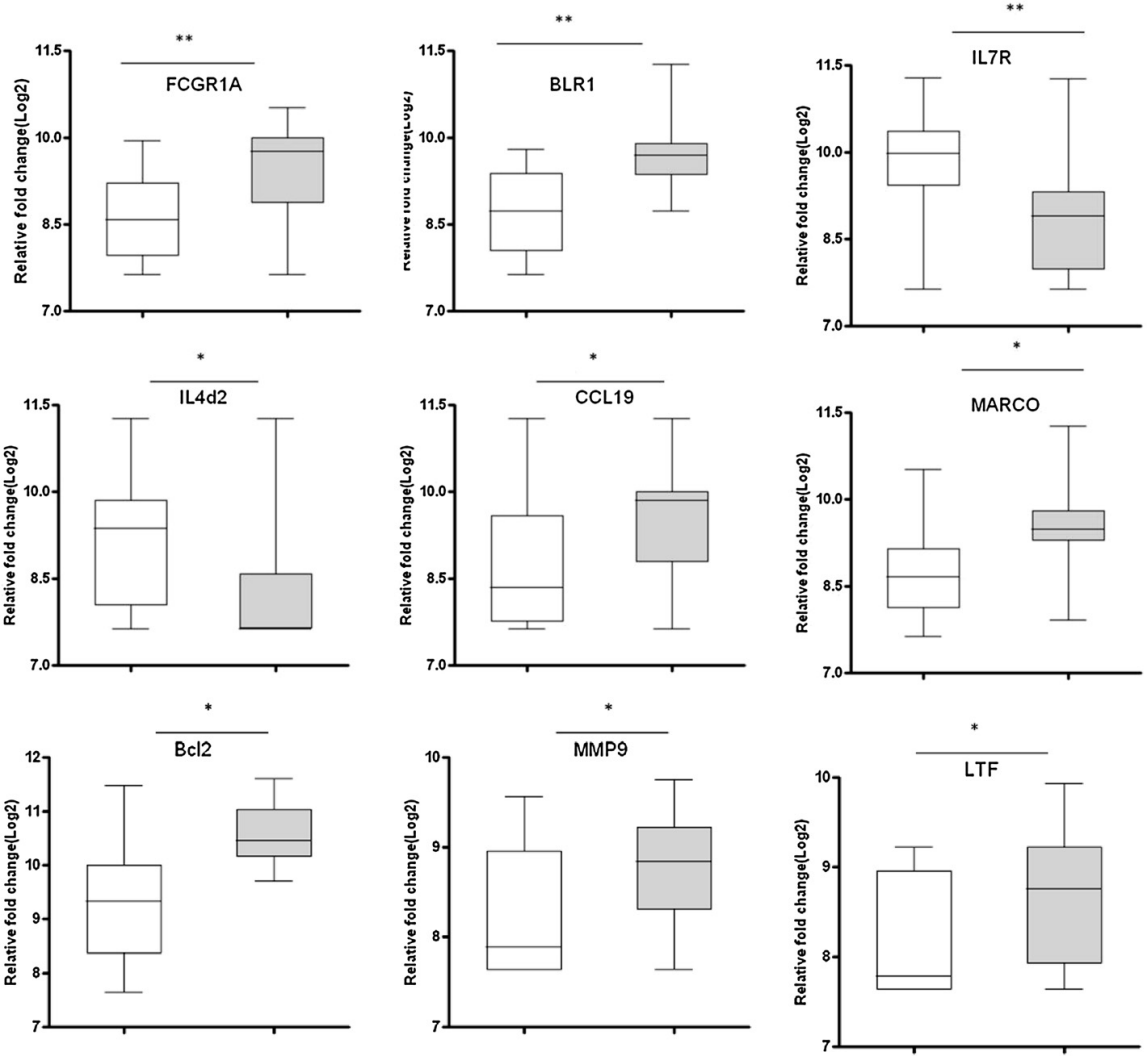
**Gene expressions in household contacts and TB cases.** Box plots are shown where the horizontal lines indicate medians of household contacts (white bars) and TB cases (grey bars) and the lower and upper edge of each boxes indicate the 25th and 75th percentiles, respectively. Data were analysed using the non-parameteric Mann- Whitney test with p-values indicating significant differences after transformation of Log2 values. *P < 0.05; **P < 0.001.

RNA samples from the 24 TB patients and 23 healthy household contacts were analysed with dcRT-MLPA and significant gene expression differences were observed between these two groups. The gene expression levels of *BLR-1, MARCO, CCL-19, MMP-9, LTF, Bcl-2, and FCγR1A* were statistically higher in TB patients than contacts (p < 0.05), whereas the expression levels of IL*4*δ*2* and *IL7R* were statistically higher in healthy contacts than TB cases (p < 0.05) (Figure 1).

**Figure 2:**
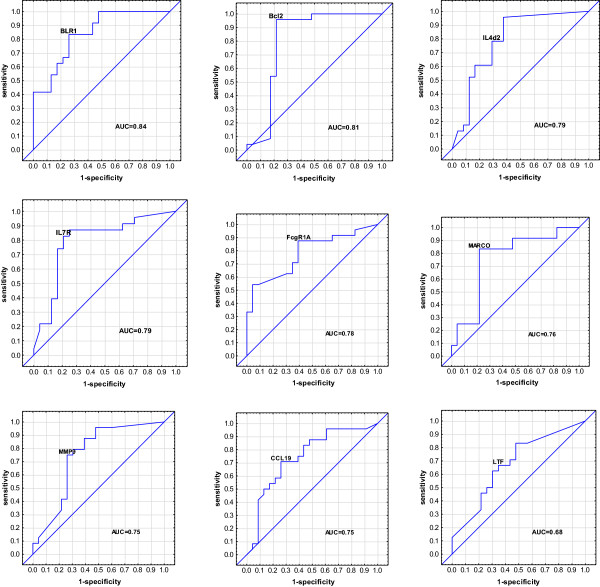
**Receiver operator characteristics curves showing the accuracies of individual genes in discriminating between active TB cases and household contacts.** Receiver operator characteristic (ROC) curves for the accuracies of single analytes to ifferentiate between active TB and household contacts. AUC = Area under the curve.

**Figure 3:**
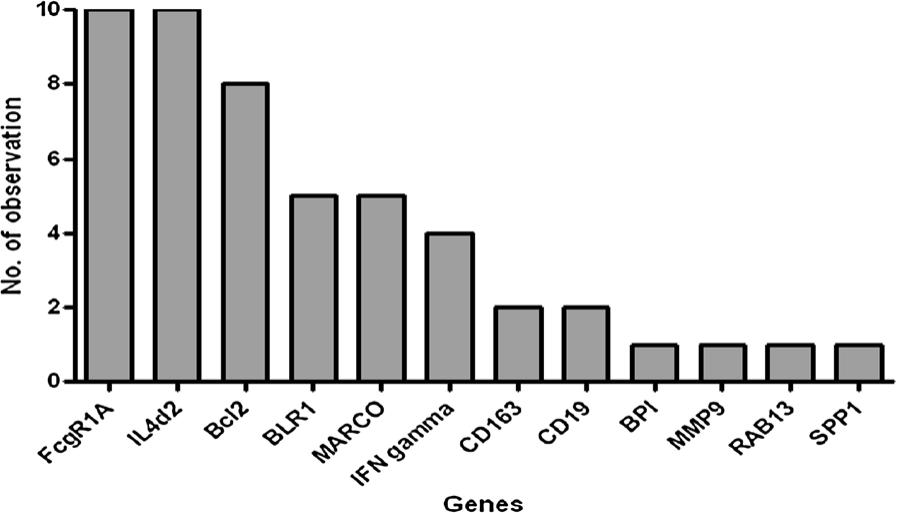
**Frequency of individual genes in top 10 models for discriminating between active TB cases and household contacts.** The columns represent the number of inclusions of individual markers into the most accurate five-analyte models by general discriminant for discriminating between active pulmonary TB cases and contacts.

These most accurate single gene markers that differentiated TB cases and contacts were *BLR1, Bcl2, IL4d2, IL7R, FcgR1A, MARCO, MMP9, CCL19,* and *LTF* with area under the curves (AUCs) of 0.84, 0.81, 0.79, 0.79, 0.78, 0.76, 0.75, 0.75 and 0.68, respectively (Figure 2). We did a best subsets discriminant analysis, revealing that a combination of five genes gave a better discriminatory power: the combination of *Bcl2, BLR1, MARCO, Fc*γ*R1A* and *IL4*δ*2* detected 95.65% (determined using leave-one-out cross validation) of household contacts and 91.66% of TB cases were correctly classified (Table 2). FcγR1A and IL4δ2 were the most frequently occurring markers in the GDA biomarker combinations differentiating between the TB cases and household contacts (Figure 3).

**Table 2:** General discriminate analysis of five marker combinations to discriminate active TB and household contacts

	**Household contacts**		**TB cases**				
	**Resubstitution classification**	**Leave-one-out cross**	**Resubstitution classification**	**Leave-one-out cross**	**Wilks lambda**		
**Genes**	**Matrix**	**Validation**	**Matrix**	**Validation**	**Value**	**f**	**p value**
Bcl2, BLR1, FcγR1A, R1A, IFNγ, IL4δ2	95.65	91.3	95.83	95.83	0.74	14	<0.001
Bcl2, FcγR1A, IFNγ, IL4δ2, MARCO	91.3	91.3	95.83	91.66	0.72	15.8	<0.001
Bcl2, BLR1,CD163, FcγR1A, IL4δ2	95.65	95.65	91.66	87.5	0.75	13.65	<0.001
Bcl2, BLR1, FcγR1A, IL4δ2, MARCO	95.65	95.65	95.83	91.66	0.73	14.95	<0.001
Bcl2, CD19, FcγR1A, IL4δ2, MARCO	95.65	95.65	91.66	91.66	0.75	13.21	<0.001
Bcl2, BLR1, CD19, FcγR1A, IL4δ2	91.3	91.3	95.83	87.5	0.77	11.98	0.0013
Bcl2, BPI, FcγR1A, IL4δ2, MARCO	95.65	95.65	91.66	95.83	0.72	16.11	<0.001
BLR 1, FcγR1A, IFNγ, IL4δ2, MMp9	95.65	86.95	95.83	91.66	0.75	13.46	<0.001
BLR 1, FcγR1A, IFNγ, IL4d2, RAB13	91.3	86.95	95.83	91.66	0.76	13.13	<0.001
BLR2, FcγR1A, IL4δ2, MARCO, SPP1	95.65	95.65	95.83	87.5	0.71	16.7	<0.001

Percentage indicates the proportion of groups discriminated using the combination of markers; and f is the measure of fit.

### Gene expression of LTBI household contacts

**Figure 4:**
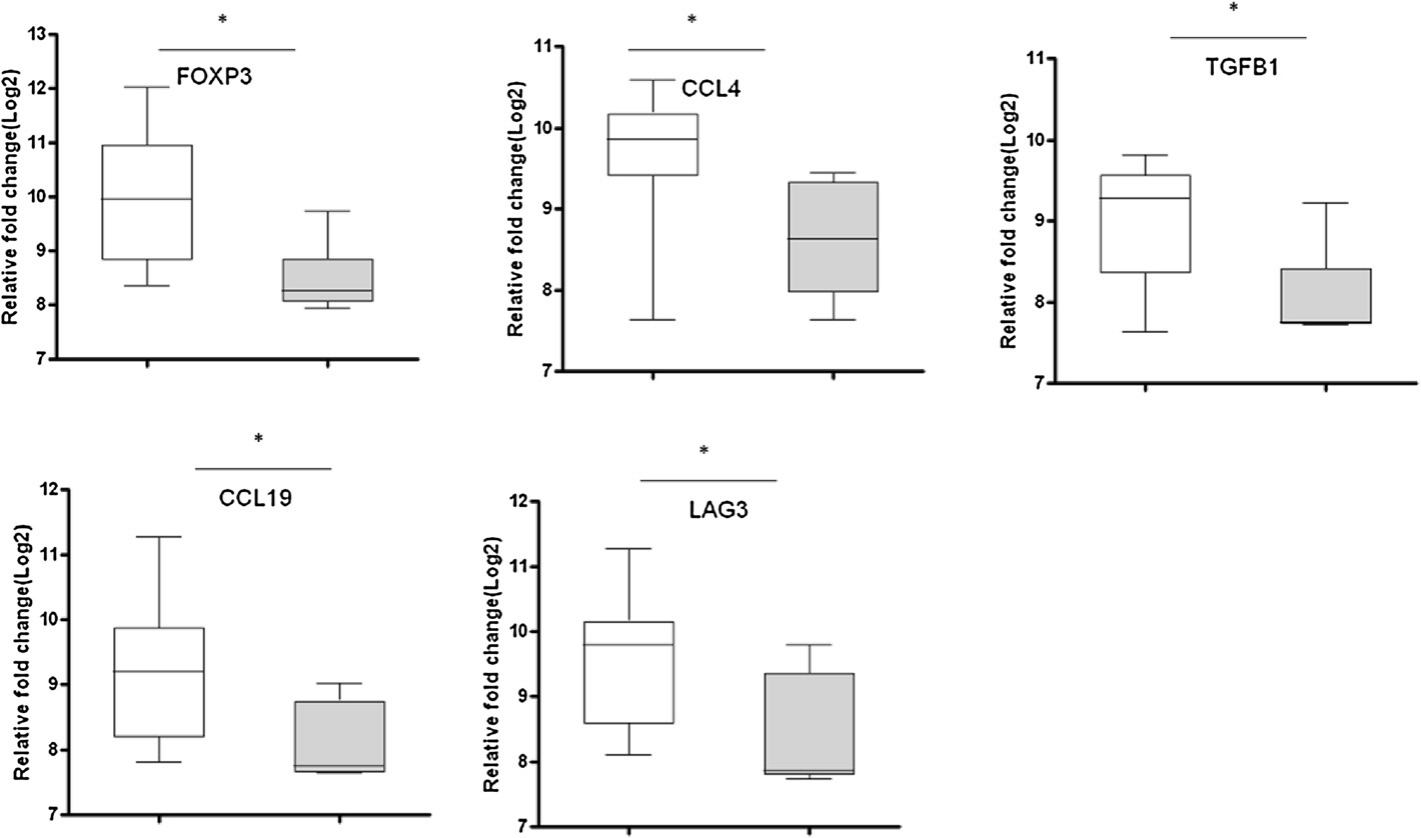
**Gene expression in Quantiferon**^**+ **^**and Quantiferon**^**- **^**household contacts.** Box plots are shown with the horizontal lines indicating median levels of Quantiferon^+^ (white bars) and Quantiferon^-^ (grey bars) household contacts. The lower and upper edge of each box indicates the 25th and 75th percentiles, respectively. Data were analysed using nonparametric Mann–Whitney test with p-values indicating significant differences after transformation of data to Log2 values. *P < 0.05.

We further classified the household contacts into LTBI and uninfected groups using the QFT test to assess the effect of LTBI on the expression level of different genes. The expression levels of *Foxp3, CCL19* and *TGFβ* were significantly higher (p < 0.05) in QFT^+^ than QFT^-^ contacts (Figure 4).

**Figure 5:**
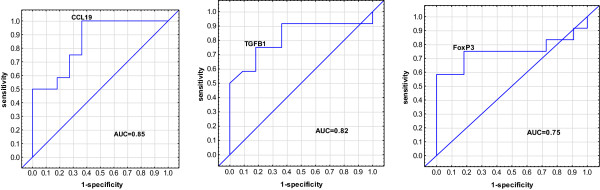
**Receiver operator characteristics curves showing the accuracies of individual genes in discriminating between LTBI and uninfected household contacts.** Receiver operator characteristic (ROC) curves for the accuracies of single analytes to differentiate between LTBI and uninfected household contacts. AUC = Area under the curve.

**Figure 6:**
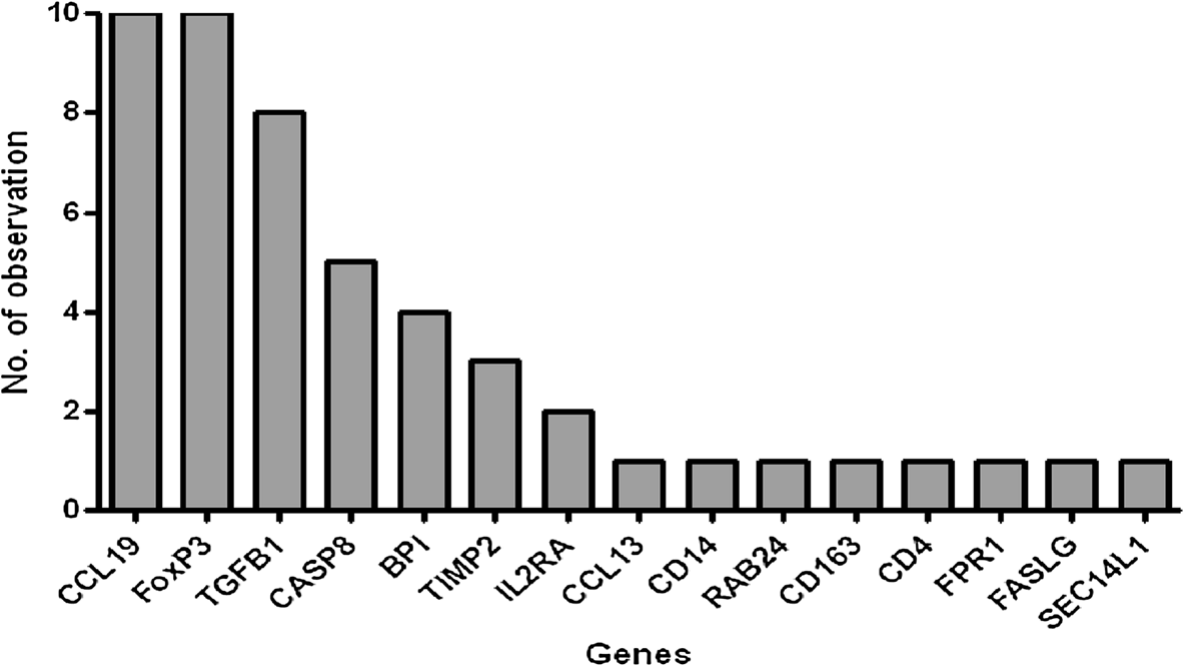
**Frequency of individual genes in top 10 models for discriminating between LTBI and uninfected household contacts.** The columns represent the number of inclusions individual markers into the most accurate five-analyte models by general discriminant for discriminating between QFT^+^ and QFT^-^ contacts.

The most accurate single gene markers that differentiated QFT^+^ and QFT^-^ contacts were *CCL19, TGFβ1, and Foxp3* with AUCs of 0.85, 0.82, and 0.75 respectively (Figure 5). A best subsets discriminant analysis (GDA) of the data indicated that optimal discrimination of LTBI and uninfected household contacts could be achieved with combinations of five variables, *BPI, CCL19, Foxp3, FPR1* and *TGFβ1.* A combination of these genes detected 90.9% QFT^-^ household contacts using leave-one-out cross validation, and detected 91.6% of QFT^+^ (Table 3). *FoxP3 and CCL19* were the most frequently occurring markers in the GDA biomarker combinations differentiating between LTBI and uninfected household contacts (Figure 6).

**Table 3:** **General discriminate analysis of five marker combinations to discriminate LTBI (QFT**^**+**^**) and uninfected (QFT **^**-**^**) household contacts**

	**QFT negative**		**QFT positive**				
	**Resubstitution classification**	**Leave-one-out cross**	**Resubstitution classification**	**Leave-one-out cross**	**Wilks lambda**		
**Genes**	**Matrix**	**Validation**	**Matrix**	**Validation**	**Value**	**f**	**p value**
BPI, CASP8, CCL19 and TGFβ1	90.9	81.8	91.6	91.6	0.84	17.45	0.093
BPI, CCL19, FOXP3, TGFβ1 and TIMP2	90.9	81.8	83.3	83.3	0.74	17.67	0.027
CASP8, CCL13, FOXP3 and TGFβ1	81.8	81.8	91.6	91.6	0.86	2.74	0.116
CCL19, CD14, FOXP3, IL2RA and TIMP2	90.9	90.9	83.3	83.3	0.62	10.22	0.005
CASP8, CCL19, FOXP3, RAB24 and TIMP2	81.8	81.8	91.6	91.6	0.92	1.54	0.23
CASP8, CCL19, CD163, FOXP3 and TGFβ1	90.9	81.8	91.6	91.6	0.92	1.53	0.23
CCL19, CD4, FOXP3, IL2RA and TIMP2	90.9	90.9	83.3	83.3	0.63	9.76	0.006
BPI, CCL19, FOXP3, FPR1 and TGFβ1	90.9	90.9	91.6	91.6	0.6	11.08	0.004
CASP8, CCl19, FASL, FOXP3 and TGFβ1	90.9	90.9	83.3	83.3	0.96	0.55	0.46
BPI, CCL19, FOXP3, SEC14L1 and TGFβ1	81.8	81.8	91.6	83.3	0.74	5.85	0.03

Percentage indicates the proportion of groups discriminated using the combination of markers; and f is the measure of fit.

## Discussion

Quantitative changes in gene expression could potentially be used as biomarkers to classify the different clinical outcomes of MTB exposure, with potential future applications in the evaluation of new drugs and vaccines. A recent study by Kaforou M. et al. [19] reported that blood transcriptional signatures distinguished TB from other conditions prevalent in HIV-infected and -uninfected African adults. We tested expression of 45 genes aimed at characterizing unique gene expression profiles in view of the complexity of the infection process and outcomes of MTB exposure and infection. This approach emphasizes the discriminatory abilities of single biomarkers, but more importantly combinations of biomarkers. In this study we used a dcRT-MLPA technique to simultaneously identify multiple genes that are differentially expressed in TB cases and their contacts and identify nine genes that were differentially expressed between TB cases and their contacts. The dcRT-MLPA technology fulfils a biomarker discovery niche between unbiased approaches such as whole transcriptome analysis and targeted analysis by qRT-PCR and enables cost-effective biomarker discovery in large field-studies with widely available laboratory equipment.

The expression levels of *FcγR1A, LTF, BLR1, MARCO, CCL-19, MMP-9, CCL4,* and *Bcl2* in whole blood was significantly higher elevated in TB patients than among contacts, whereas the expression of *IL4*δ*2* and *IL-7R* were significantly higher in healthy contacts as compared to TB cases. The higher expression of *FcγR1A* and *LTF* in TB patients has been reported previously in Germany [12] with microarray analysis of PBMCs from TB patients and healthy donors and in Gambia and Paraguay with MLPA [15] and a recent study showed expression of significantly higher level expression of *FcγR1A* in participants with active TB than in those with LTBI before treatment regardless of HIV status or genetic background [20]. *FcγR1A* and *LTF* are essential components of antimicrobial defenses and blocking of induction of *FcγR1A* is one major target for the survival strategy of MTB [21]. LTF, in addition to regulating iron uptake and utilization, modulates both the innate and adaptive immune response and the potential of LTF as an adjuvant for BCG vaccination has been considered [22]. Another study in a murine model also showed that susceptibility to TB could be reduced by avoiding overload of iron using LTF [23].

*BLR1 (CXCR5)* encodes a chemokine receptor and the higher expression of this gene in TB patients might help in sustaining the expression of its ligand CXCL13, which in turn attracts B cells. A role of B cells in immunity against TB has been observed in some studies [24,25]. The higher level of *CCL19* in TB patients could be due to active infection where a number of crucial cells including macrophages and T cells are recruited to contain infection. Different in vivo and in vitro studies indicate that MTB infection of human monocyte derived macrophages, alveolar macrophages, and CD4^+^ T cells induce upregulation of chemokine receptors and their ligands [26-28].

The higher level of *MMP9* and *MARCO* in TB infections is in line with other previous reports, revealing higher level of MMP-9 in TB cases where it facilitates early dissemination of MTB with subsequent recruitment of macrophages, induction of Th1 type immunity and granuloma formation [29,30]. *MARCO* is a phagocytic receptor and MTB uses different receptors for entry into macrophages. Previous work in mouse models also showed upregulation of *MARCO* genes after BCG infection [31] and a low proinflammatory response of MARCO^-/-^ mice in response to infection with virulent MTB [32].

The remaining other genes that had discriminatory power were *Bcl-2* and *IL-4*δ*2. Bcl-2* is an anti-apoptotic gene and in this study, its expression was higher in TB patients. Apoptosis and autophagy likely participate in elimination of infected cells without releasing viable bacteria. Previous studies in Ethiopia and Gambia [15,33] indicate up regulation of apoptotic genes in TB patients but we did not observe these findings in our study. However, the higher expression of Bcl2 which we observe shown here instead could be part of a pathogen survival strategy. Previous studies also showed that MTB or its products can inhibit apoptosis [34]. We found the expression of *IL4*δ*2* to be higher in contacts than in TB patients. *IL4*δ*2* is a recently described splice variant of IL4 which inhibits IL4 activity. LTBI individuals expressed high levels of Th1 cytokines and the *IL4* antagonist *IL4*δ*2,* and individuals with a high IL4δ2/IL4 ratio were reported being capable of controlling MTB infection [35].

The expression of *CCL19, TGFβ1*, and *Foxp3* discriminates LTBI and uninfected contacts with higher expression of all genes in IGRA positive latently MTB infected individuals. Their higher expression might be due to recent MTB infection, resulting in immune activation and recruitment of immune cells. *CCL19* is critical for recruitment of activated immune cells. Increased expression of regulatory molecules could help regulating exacerbated immune activation and preventing excessive inflammation and resulting causing immunopathology. Regulatory molecules like Foxp3 and TGF*β*1 indeed have been reported to regulate immune responses during infection thereby preventing excessive inflammation and tissue damage [36]. Another study also showed activation and expansion of both T effector cells and Foxp3 (+) T reg populations early in MTB infection. IL2 induces expression of both effector and regulatory T cells and confers resistance against severe MTB infection [37].

## Conclusion

In conclusion, active TB cases versus healthy TB contacts, as well as LTBI versus uninfected healthy TB patient contacts could be accurately differentiated using expression of single genes and particularly multi-component - combinations of genes with improved discriminatory power. Hence, our findings deserve further validation in larger studies and prospective cohorts.

## Competing interests

The authors declare that they have no competing interests.

## Authors’ contributions

AM involved in study design, laboratory work, data collection and MLPA analysis/interpretation and drafted the manuscript. AL involved in data collection, laboratory work, and MLPA analysis/interpretation and drafted the manuscript. YB involved in laboratory work and data collection. SK involved in study design and write up. MK involved in data analysis. AA involved in study design, data analysis and write up. MCK participated in study design, data analysis and write up. TO participated in study design, data analysis and write up. RH involved in study design, data analysis and write up. GW involved in study design, data analysis and write-up. All authors read and approved the final manuscript.

## Pre-publication history

The pre-publication history for this paper can be accessed here:

http://www.biomedcentral.com/1471-2334/14/257/prepub
